# Differences between sporadic and MEN related primary hyperparathyroidism; clinical expression, preoperative workup, operative strategy and follow-up

**DOI:** 10.1186/1750-1172-8-50

**Published:** 2013-04-01

**Authors:** Bas A Twigt, Anouk Scholten, Gerlof D Valk, Inne HM Borel Rinkes, Menno R Vriens

**Affiliations:** 1 Department of Surgical Oncology and Endocrine Surgery, University Medical Center, Hp.nr. G04.228, Heidelberglaan 100, Utrecht, 3584CX, the Netherlands; 2 Department of Endocrinology, University Medical Center, Utrecht, the Netherlands

**Keywords:** Parathyroid, Familial hyperparathyroidism, PTH, Multiple endocrine neoplasia type 1, Multiple endocrine neoplasia type 2, Hypercalcemia

## Abstract

**Background:**

Primary hyperparathyroidism (PHPT) is most commonly sporadic (sPHPT). However, sometimes PHPT develops as part of multiple endocrine neoplasia (MEN) type 1 or 2A. In all, parathyroidectomy is the only curative treatment. Nevertheless, there are important differences in clinical expression and treatment.

**Methods:**

We analyzed a consecutive cohort of patients treated for sporadic, MEN1-related, and MEN2A-related PHPT and compared them regarding clinical and biochemical parameters, differences in preoperative workup, operative strategies, findings, and outcome.

**Results:**

A total of 467 patients with sPHPT, 52 with MEN1- and 16 with MEN2A-related PHPT were analyzed. Patients with sPHPT were older, more often female and had higher preoperative calcium and parathyroid hormone levels, when compared with MEN1 and MEN2A patients. Minimally invasive parathyroidectomy (MIP) was performed in 367 of 467 sPHPT patients (79%). One abnormal parathyroid was found in 426 patients (91%). Two or more in 35 patients (7%). In six patients (1%) no abnormal parathyroid gland was retrieved. Of 52 MEN1 patients, eight (15%) underwent a MIP and 44 patients (85%) underwent conventional neck exploration (CNE); with resection of fewer than 3½ enlarged glands in 21 patients (40%), subtotal parathyroidectomy (SPTX, 3-3½ glands) in seventeen (33%) and total parathyroidectomy with autotransplantation (TPTX) in six (12%). Eleven patients (21%) had persistent disease, 29 (56%) recurrent PHPT and nine (17%) permanent hypoparathyroidism, mostly after TPTX. Of 16 MEN2A patients, six (38%) underwent MIP, four (25%) CNE and six (38%) selective resection of the enlarged gland(s) during total thyroidectomy. Three patients (19%) suffered from persistent PHPT and two (13%) developed recurrent disease.

**Conclusions:**

Sporadic PHPT, MEN1- and MEN2A-related PHPT are three distinct entities as is reflected preoperatively by differences in gender, age at diagnosis and calcium and PTH levels.

MEN2A patients are very similar to sPHPT with respect to operative approach and findings. MIP is the treatment of choice for both. MIP has low rates of persistent and recurrent PHPT and a low complication rate. The percentage of multiglandular disease and recurrences are significantly higher in MEN1 patients, demonstrating the need for a different approach. We advocate treating these patients with CNE and SPTX.

## Background

The etiology of primary hyperparathyroidism (PHPT), a common endocrine disease, is most frequently sporadic and non-familial (sPHPT) [1]. A significant proportion of patients, however, develop PHPT as part of the familial syndromes multiple endocrine neoplasia (MEN) type 1 or 2A [2,3]. Major features of MEN1 are endocrine tumors of the parathyroid, pituitary, and pancreas. Minor features consists of bronchial and thymic tumors [4,5]. MEN2A is associated with medullary thyroid cancer, PHPT, and pheochromocytoma [6]. In all cases of PHPT, parathyroidectomy is the only curative treatment that resolves symptoms and metabolic complications and thus improves quality of life, in both symptomatic and asymptomatic patients.

Clinical features that differentiate between patients with sporadic PHPT and MEN-related PHPT are: age of onset, female to male ratio, severity of bone involvement, family history and related endocrine neoplasias [7-9]. Once PHPT and its setting are diagnosed, the course of the disease and its treatment will change the perspective for both surgeon and patient considerably.

However, most of the current literature analyzes data on surgical treatment of PHPT without making any such distinction to this profound difference in etiology [9-11]. Although hypercalcemia might be the first clinical parameter to be discovered in all three, we strongly believe, these are very distinct and different entities, requiring a different approach.

In a population based cohort of patients treated for PHPT, we evaluated the frequency and causes (number of affected glands) of sporadic, MEN1- and MEN2A-related PHPT, as well as the differences in their clinical presentation, preoperative workup and operative strategies, findings and outcome. We sought to determine whether, with optimal surgical strategies for each subgroup, a comparable outcome (low persistent and recurrence rates) with equally low complication rates (hypoparathyroidism and recurrent laryngeal nerve injury) could be obtained.

## Patient and methods

We retrospectively analyzed the records of a consecutive cohort of patients treated for sPHPT in one geographical region of The Netherlands between 1994 and 2009, comprising one academic center and three affiliated hospitals. All patients were symptomatic. The diagnosis PHPT was established biochemically by a serum calcium level greater than 10.20 mg/dL (>2.55 mmol/L) and/or a serum ionized calcium greater than 5.28 mg/dL (>1.32 mmol/L) combined with an increased, greater than 65 pg/mL (>6.5 pmol/L), or not suppressed plasma parathyroid hormone (PTH) level. In a few patients calcium levels were normal, but an increased renal calcium excretion combined with an elevated PTH level was affirmative for PHPT [12-14]. In addition, all patients with PHPT from the MEN1 and MEN2 database at the University Medical Center Utrecht (UMCU), The Netherlands were analyzed. The MEN1 database includes patients diagnosed with PHPT between 1967 and 2009. Patients were included in the MEN1 database if they had genetically proven MEN1, or three out of five manifestations of MEN1 or one out of five manifestations and a first-degree family member. Gene testing (mutation analysis) was performed in very young patients with PHPT, PHPT in combination with possible MEN1 manifestations, or a MEN- positive family history [15]. From the MEN2 database, patients diagnosed with PHPT between 1979 and 2009 were selected. MEN2A was defined in case of a MEN2A germline mutation. Patients with MEN1 and MEN2A were included if they had biochemical evidence of PHPT as stated above or enlarged parathyroid glands while undergoing a total thyroidectomy. Since the UMCU is a tertiary referral center, also patients who were initially treated at another institution and later referred to our institution were included. All patients gave ethical consent for their information to be held anonymously in our database and to be used for future retrospective analysis.

Preoperative localizing studies were used in sPHPT and MEN2A patients and included ultrasonography (US), computed tomography (CT) and/or technetium-99m-sestamibi-scintigraphy (MIBI). The preoperative diagnostic work up differed between hospitals and evaluated over time. Presently, our preferential preoperative work up consists of MIBI and US. Depending on the results of the preoperative localization studies, sPHPT and MEN2A patients were subsequently operated in a preferentially minimally invasive approach [16]. MIP was defined as a small (3cm) incision over the suspected adenoma as guided by preoperative localization (two concordant preoperative imaging techniques), whereas a unilateral approach involves a larger incision and exposure plus systematic exploration of the entire area of interest on one side (based on one positive preoperative imaging). Both inferior and superior parathyroid glands will have to be identified using this approach. In case of no visualization of an enlarged gland or discordant imaging techniques a conventional neck exploration (CNE) was performed. Preoperative imaging for MEN1 patients is not part of our policy, although many patients underwent preoperative imaging studies prior to referral to our surgical department. In subtotal parathyroidectomy (SPTX), 3–3½ parathyroid glands were resected during a CNE after identification of all parathyroid glands. In total parathyroidectomy (TPTX), four glands were resected and one (partial) gland was used as a graft for autotransplantation into the brachioradial muscle of the nondominant forearm. The autotransplantation was performed during the same operation, using fresh parathyroid tissue.

Intraoperative PTH measurements (IOPTH) and/or intraoperative frozen section analysis, to verify removal of aberrant parathyroid tissue, were carried out in a routine fashion whenever a minimally invasive parathyroidectomy (MIP) was performed. A significant drop of more than 50% from the highest of either preoperative baseline or preexcision level at 10 minutes after hyperfunctioning parathyroid gland(s) excision indicates surgical cure and predicts postoperative normocalcemia [17,18].

Surgical cure was defined as a normalization of serum (ionized) calcium and PTH levels for a period of at least six months after the surgical procedure. Persisting hypercalcemia or renewed hypercalcemia within the first six months after surgery was considered indicative of surgical failure. Hypercalcemia after a period of six month of postoperative normocalcemia was defined as recurrent disease. The findings of all operations necessary to achieve normocalcemia were taken into account when determining the cause of PHPT. Extirpation of a single enlarged parathyroid gland with subsequent normalization of serum calcium was defined as single gland disease. Retrieval of more than one enlarged parathyroid gland leading to normocalcemia was defined as multiglandular disease (MGD). Multiglandular hyperplasia was defined as the situation when all four glands appeared abnormal. Hypoparathyroidism and nerve damage were considered complications of surgery. Permanent hypoparathyroidism was defined as a serum ionized calcium of less than 4.60 mg/dL (<1.15 mmol/L) and/or total calcium of less than 8.48 mg/dL (<2.12 mmol/L), persisting beyond the first six months after surgery and requiring substitution with calcium and an active form of vitamin D.

To get insight into PHPT in MEN1 and MEN2A and their difference with respect to sPHPT, we evaluated clinical and biochemical parameters, differences in preoperative workup, operative strategies, and findings.

All continuous variables were reported as median (range). Mann–Whitney U test and Independent-Samples T Test were used for two-group comparison of continuous variables and Chi squared test for analysis of categorical data. Statistical analysis was performed using SPSS version 15.0 (SPSS, Inc., Chicago, IL). Statistical significance was established at p<0.05.

## Results

A total of 535 patients were analyzed. The cohort consists of 467 patients with sPHPT, 52 with MEN1- and 16 with MEN2A-related PHPT. Patient characteristics are summarized in Table 1. Gender, age, preoperative calcium and PTH levels were significantly different among groups. In the sPHPT group, there were more females, patients were older and preoperative calcium and PTH levels were higher compared with the MEN1 and MEN2A patients (p<0.001 Chi^2^, p<0.001 Independent-Samples T Test and p=0.012 Mann–Whitney U test, respectively). Clinical complaints as lethargy and renal stones were not significantly different between sPHPT patients and MEN1 and MEN2A patients (p=0.184 and p=0.06 versus p=0.22 and p=0.59 Chi^2^, respectively).

**Table 1:** Characteristics of Primary Hyperparathyroidism in Sporadic, MEN1 and MEN2A Patients

**Characteristics**	**Sporadic pHPT, *n *= 467**	**MEN1-pHPT, *n *= 52**	**MEN2A-pHPT, *n *= 16**
Female, n	357 (76%)	33 (63%)	9 (56%)
Age, y, median (range)	63 (20 to 88)	33 (11 to 62)	39 (20 to 66)
Symptoms at first presentation, n	467 (100%)	42 (81%)	12 (75%)
Fatigue	188 (40%)	16 (31%)	4 (25%)
Renal stones	115 (25%)	14 (27%)	3 (19%)
Osteoporosis	73 (16%)	0	4 (25%)
Gastrointestinal symptoms	67 (14%)	7 (14%)	0
Neuropsychiatric	42 (9%)	7 (14%)	1 (6%)
Preoperative serum level, mean (range)			
Ionized calcium, mg/dL	6.76 (4.60 to 7.40)	5.56 (4.44 to 6.44)	5.4 (5.32 to 5.68)
Calcium, mg/dL	11.56 (10.12 to 22.20)	-	-
Parathyroid hormone, pg/mL	219 (10 to 3097)	78 (16 to 191)	89 (52 to 249)
Imaging modality, n			
Ultrasonography	399 (85%)	30 (57%)	10 (63%)
Computed tomography	317 (68%)	13 (25%)	8 (50%)
Technetium-99m-sestamibi-scintigraphy	206 (44%)	12 (23%)	8 (50%)
Number of used imaging modalities, mean	1.97	1.06	1.63

Abbreviations: *pHPT*, primary hyperparathyroidism; *MEN1*, multiple endocrine neoplasia type 1; *MEN2A*, multiple endocrine neoplasia type 2A.

The average number of preoperative imaging was similar in the sPHPT and MEN2A group (mean number of used imaging modalities 1.97 and 1.63 respectively), but higher when compared with MEN1 patients (mean number of used imaging modalities 1.06).

The operative findings and postoperative course, as well as the complications for each group are described in Tables 2 and 3.

**Table 2:** Surgery for Primary Hyperparathyroidism in Sporadic, MEN1 and MEN2A

**Characteristics**	**Sporadic pHPT, *n *= 467**	**MEN1-pHPT, *n *= 52**	**MEN2A-pHPT, *n *= 16**
Initial operation, n			
Minimally invasive parathyroidectomy	328 (70%)	5 (10%)	6 (38%)
Minimally invasive parathyroidectomy converted to conventional neck exploration	39 (8%)	3 (6%)	0
Conventional neck exploration	100 (21%)	44 (84%)	10 (62%)
Subtotal parathyroidectomy, n	-	38 (73%)	-
Total parathyroidectomy, n	-	6 (12%)	-
Number of operations, n			
One procedure	435 (93%)	26 (50%)	13 (81%)
Two procedures	31 (7%)	17 (33%)	1 (6%)
Three or more procedures	1 (<1%)	9 (17%)	2 (13%)
Mean number of operations	1.07	1.85	1.29
Cumulative operative findings, n			
No adenoma found	6 (1%)	-	1 (6%)
1 enlarged gland	426 (91%)	17 (33%)	13 (81%)
Solitary (adenoma, hyperplasia)	422 (90%)	17 (33%)	13 (81%)
Carcinoma	4 (1%)	-	-
> 1 enlarged glands	35 (7%)	35 (56%)	2 (13%)
2 enlarged glands	26 (6%)	12 (23%)	2 (13%)
3 enlarged glands	8 (2%)	17 (33%)	-
> 3 enlarged glands or hyperplasia	1 (<1%)	6 (12%)	-

Abbreviations: *pHPT*, primary hyperparathyroidism; *MEN1*, multiple endocrine neoplasia type 1; *MEN2A*, multiple endocrine neoplasia type 2A. Subtotal parathyroidectomy; (less than) SPTX. Total parathyroidectomy; TPTX.

**Table 3:** Outcome of Surgery for Primary Hyperparathyroidism in Sporadic, MEN1 and MEN2A

**Characteristics**	**Sporadic pHPT, *n *= 467**	**MEN1-pHPT, *n *= 52**	**MEN2A-pHPT, *n *= 16**
Persistent disease, n			
After first procedure	31 (7%)	11 (21%)	3 (19%)
After second procedure	6 (1%)	4 (8%)	1 (6%)
Recurrent disease, n			
After first procedure	3 (<1%)	28 (54%)	2 (13%)
After second procedure	-	12 (24%)	1 (6%)
Complications, n			
Recurrent laryngeal nerve injury	3 (<1%)	1 (2%)	1 (6%)
Hypocalcemia	1 (<1%)	10 (19%)	2 (13%)

Abbreviations: *pHPT*, primary hyperparathyroidism; *MEN1*, multiple endocrine neoplasia type 1; *MEN2A*, multiple endocrine neoplasia type 2A.

### sPHPT

Of 467 patients with sPHPT, treated in the UMCU or in one of three regional teaching hospitals, 367 patients (79%) were scheduled for a MIP. The remaining 100 patients underwent a planned CNE. In 39 patients (8%) a MIP procedure was intraoperatively converted to a CNE. In 18 of these patients, the minimal invasive approach provided insufficient exposure to enucleate a correctly localized adenoma. In one patient the adenoma was not found. In the other 20 patients, the preoperative imaging was not consistent with the intraoperative findings. The surgical success rate after primary surgery was 93% (n=435). Hypercalcemia persisted after the first operation in 31 patients (7%). The persistence rate in patients with IOPTH measurement was 4%. The cumulative surgical success rate, including an early second operative procedure, was 99% (n=461). Normocalcemia resulted from removing one abnormal parathyroid gland in 426 patients (91%). Two or more abnormal glands were removed in 35 patients (7%), while four gland hyperplasia was the observed cause of PHPT in one patient. In six patients (1%) no abnormal parathyroid gland was retrieved and thus hypercalcemia persisted. Four patients developed recurrent hypercalcemia. Parathyroid carcinoma was diagnosed in four patients. The median follow-up was two years (range 1–15 years). Three patients sustained permanent recurrent laryngeal nerve damage and one patient became permanent hypocalcemic.

### MEN1

Fifty-two patients underwent primary surgery for PHPT either at the UMCU (n=36) or another affiliated hospital (n=16). Eight patients (15%) underwent a MIP, twenty-one underwent less than SPTX (<SPTX), seventeen underwent SPTX and six underwent TPTX. In three patients a MIP procedure was intraoperatively converted to a CNE and TPTX due to inadequate drop of IOPTH levels. Eleven patients (21%) had persistent disease; nine patients after <SPTX (31%), one after SPTX (7%) and one after TPTX (17%). Twenty-eight patients (54%) developed recurrent PHPT, after a median time to recurrence of 8.0 years after <SPTX (56%), and after a median time of 13.0 years after SPTX (65%). None of the patients who underwent TPTX had recurrence. After primary surgery, ten patients (19%) developed permanent hypoparathyroidism; 7% after <SPTX, 25% after SPTX and 67% of the patients who underwent TPTX. One patient had a permanent recurrent laryngeal nerve injury, after multiple operations for persistent and recurrent PHPT.

### MEN2A

Sixteen MEN2A patients underwent primary surgery for parathyroid disease between 1979 and 2010. Eleven operations were carried out at the UMCU and five in affiliated hospitals. Ten patients were operated in varying years after a previous total thyroidectomy. Six patients of these underwent MIP and four patients underwent CNE (n=3 one gland resected, n=1 two glands resected). In the other six patients selective resection of the enlarged gland(s) was performed during total thyroidectomy for medullary thyroid carcinoma (n=1 no glands resected because none were found, n=4 one gland resected, n=1 two glands resected). None of our MEN2 patients underwent a parathyroidectomy before they underwent a thyroidectomy. Thirteen patients were initially cured after the primary operation. Three patients suffered from persistent PHPT, two patients developed recurrent disease. The mean overall follow-up after primary surgery was 9 years (range 5–27 years). After MIP, one patient had persistent PHPT, but no one developed recurrent PHPT during five years of follow-up. Five patients had hypoparathyroidism, due to inadvertent damage to parathyroid glands during total thyroidectomy.

The percentage of operations started as a minimally invasive operation was higher in the sPHPT group when compared with the MEN populations (p <0.001, chi^2^). The average number of operations was higher in MEN patients when compared with the sPHPT population (p<0.001, chi^2^); between MEN1 and MEN2A we could not demonstrate a significant difference. The number of patients with MGD was the highest in the MEN1 group (p<0.001, chi^2^).

## Conclusions

### Main findings

**Figure 1:**
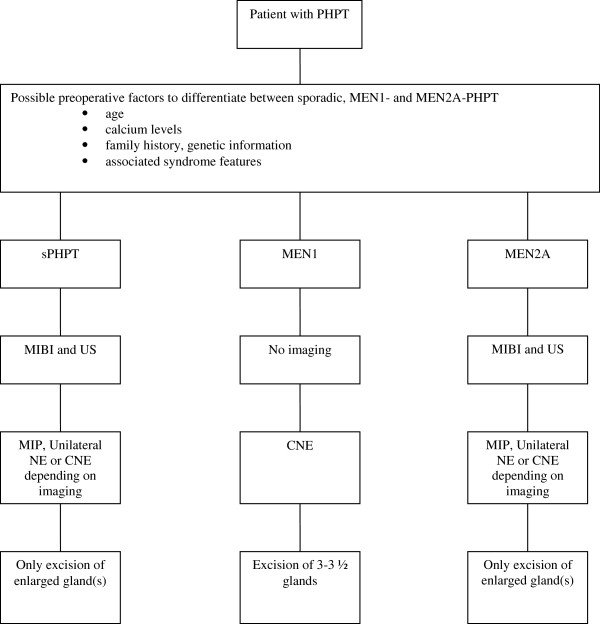
**Treatment algorithm for patient with PHPT.** PHPT primary hyperparathyroidism, MEN1 multiple endocrine neoplasia type 1, MEN2A multiple endocrine neoplasia type 2A, sPHPT sporadic primary hyperparathyroidism, MIBI technetium-99m-sestamibi-scintigraphy, US ultrasound, MIP minimally invasive parathyroidectomy, NE neck exploration, CNE conventional neck exploration.

According to our study and previous literature, sporadic PHPT, MEN1- and MEN2A-related PHPT are three distinct entities as is reflected preoperatively by differences in gender, age at diagnosis, and preoperative calcium and PTH levels [7-9]. Clearly this leads to a distinct algorithm regarding the preoperative workup and operative strategy (Figure 1). We found no difference in the prevalence of clinical symptoms, in agreement with previous studies [9,19].

### Our data compared wtih the literature

#### sPHPT

As others have demonstrated operative findings may be a function of the operative approach; a CNE leads to removal of more parathyroid glands and thus a higher percentage of multiglandular disease [20,21]. In our series the frequency of solitary adenomas observed is higher than historically reported [22]. The extent of the preoperative workup influences the number of observed solitary adenomas. In case of two concordant imaging studies we advocate to perform a MIP, if there is only one positive study a unilateral exploration and if all imaging studies are negative or contradictive an upfront CNE. The use of IOPTH remains controversial and we advise not to use it in a routine fashion [23-29]. Others, however ,do show benefits of IOPTH measurement [30-32]. Especially, in patients with recurrent disease, in patients with proven or suspected multiglandular disease, as well as in patients with inconsistent preoperative imaging, IOPTH can add to decision making and improve outcome [33-35]. We included some patients with a normal calcium level. One might argue whether in these patients PHPT can be diagnosed. However, pathology examination confirmed the diagnosis. Part of these patients might only be intermittent normocalcemic or become asymptomatic patients later. Furthermore there is some evidence for a generalized target-tissue resistance to parathyroid hormone and as a result a renal tubular resistance to the action of PTH and thus increased renal calcium excretion [12-14].

#### MEN1

The percentage of MGD is significantly higher in MEN1 patients, this demonstrates the need for a different approach in this category of patients. Ninety-five percent of MEN1 patients were treated with a CNE. Some advocate to perform a TPTX [36-38]. Based on the data presented by the DutchMEN1 Study Group, who reported a genotype-phenotype correlation in MEN1-related PHPT, we have changed our surgical strategy over the last years. Part of the patients in the present study was included in the patient cohort of a previous study. Recurrence after <SPTX, in this cohort, was significantly lower in patients with nonsense or frameshift mutations in exon 2,9, and 10. This indicates that cure primarily depends on the amount of parathyroid tissue removed. As these results have to be confirmed in an independent patient population, we have not repeated this analysis in the present cohort [5]. Because TPTX frequently results in hypoparathyroidism [38-45], SPTX combined with bilateral transcervical thymectomy is now the preferred procedure in our institution, providing the best balance between cure and postoperative hypoparathyroidism [5,46]. When taken the high number of CNE into account we found the number of used imaging modalities in the MEN1 group rather high. A plausible cause might be the unawareness of referring physicians with the possibility of MEN-related PHPT and the inability to localize a solitary gland at first presentation causing more extensive preoperative imaging.

#### MEN2A

Despite different patient characteristics, MEN2A patients are very similar to patients with sPHPT with respect to their operative approach and intraoperative findings. A focused MIP is therefore the treatment of choice for PHPT in MEN2A patients [47]. MIP has low rates of persistent and recurrent PHPT and the complications are minimal. Especially patients treated in more recent years have equal rates of solitary and multiglandular disease.

### Weaknesses and strengths

Weaknesses of our study are the fairly large differences in the number of sporadic, MEN1- and MEN2A-related PHPT patients and the time period in which they were treated. Unfortunately, due to the rarity of MEN syndromes these differences are inevitable. Furthermore, treatment of all three categories has gradually changed over the years due to more refined preoperative localization techniques, IOPTH measurement and the growing awareness and understanding of their differences in pathophysiology and genotype. This implies a heterogeneous case mix. On the other hand, this does reflect the clinical practice over the past decades in many hospitals and countries. A potential confounding factor is the location of treatment. The majority of MEN patients were treated in a tertiary referral center, whereas (50%) of the sPHPT patients were treated in an affiliated hospital. However, preoperative imaging and a preferentially minimally invasive approach was the standard of care in all four hospitals. Many studies have focused on patients with sporadic and MEN-related PHPT separately. The strength of this study is the description of both phenotype, preoperative work up and surgical strategy in all three categories; offering a complete overview and a treatment algorithm.

### Conclusion

We performed a descriptive case–control study in which the different outcomes for sporadic, MEN1- and MEN2A-related primary hyperparathyroidism were assessed and possible contributing confounding factors were analyzed. In light of our findings in these three categories of patients; i.e. the significant higher number of MGD, reoperation rate and percentage of recurrent disease in MEN1 patients we advocate the treatment algorithm as outlined in Figure 1. In our opinion these findings are a corroboration to concentrate and treat MEN patients in a tertiary referral center.

## Competing interests

There are no financial or nonfinancial competing interests.

## Authors’ contributions

The work presented here was a collaboration between all authors. All authors helped to define the research theme and study design. BA Twigt and A Scholten carried out the collection of data, analyzed the data and interpreted the results. BA Twigt and A Scholten wrote the paper. MR Vriens, IHM Borel Rinkes and GD Valk helped with interpretation of the data, discussed the analysis and critically revised the article. All authors read and approved the final manuscript.
